# Diversity and evolution of transposable elements in the plant-parasitic nematodes

**DOI:** 10.1186/s12864-024-10435-7

**Published:** 2024-05-23

**Authors:** Mehmet Dayi

**Affiliations:** 1 Forestry Vocational School, Düzce University, Konuralp Campus, 81620 Düzce, Türkiye; 2 Faculty of Medicine, University of Miyazaki, Miyazaki, Japan; 3 Department of Integrated Biosciences, Graduate School of Frontier Sciences, The University of Tokyo, Chiba, 277-8562 Japan

**Keywords:** Transposable elements, Plant-parasitic nematodes, Genome evolution

## Abstract

**Background:**

Transposable elements (TEs) are mobile DNA sequences that propagate within genomes, occupying a significant portion of eukaryotic genomes and serving as a source of genetic variation and innovation. TEs can impact genome dynamics through their repetitive nature and mobility. Nematodes are incredibly versatile organisms, capable of thriving in a wide range of environments. The plant-parasitic nematodes are able to infect nearly all vascular plants, leading to significant crop losses and management expenses worldwide. It is worth noting that plant parasitism has evolved independently at least three times within this nematode group. Furthermore, the genome size of plant-parasitic nematodes can vary substantially, spanning from 41.5 Mbp to 235 Mbp. To investigate genome size variation and evolution in plant-parasitic nematodes, TE composition, diversity, and evolution were analysed in 26 plant-parasitic nematodes from 9 distinct genera in Clade IV.

**Results:**

Interestingly, despite certain species lacking specific types of DNA transposons or retrotransposon superfamilies, they still exhibit a diverse range of TE content. Identification of species-specific TE repertoire in nematode genomes provides a deeper understanding of genome evolution in plant-parasitic nematodes. An intriguing observation is that plant-parasitic nematodes possess extensive DNA transposons and retrotransposon insertions, including recent sightings of LTR/Gypsy and LTR/Pao superfamilies. Among them, the Gypsy superfamilies were found to encode Aspartic proteases in the plant-parasitic nematodes.

**Conclusions:**

The study of the transposable element (TE) composition in plant-parasitic nematodes has yielded insightful discoveries. The findings revealed that certain species exhibit lineage-specific variations in their TE makeup. Discovering the species-specific TE repertoire in nematode genomes is a crucial element in understanding the evolution of genomes in plant-parasitic nematodes. It allows us to gain a deeper insight into the intricate workings of these organisms and their genetic makeup. With this knowledge, we are gaining a fundamental piece in the puzzle of understanding the evolution of these parasites. Moreover, recent transpositions have led to the acquisition of new TE superfamilies, especially Gypsy and Pao retrotransposons, further expanding the diversity of TEs in these nematodes. Significantly, the widely distributed Gypsy superfamily possesses proteases that are exclusively associated with parasitism during nematode-host interactions.

These discoveries provide a deeper understanding of the TE landscape within plant-parasitic nematodes.

**Supplementary Information:**

The online version contains supplementary material available at 10.1186/s12864-024-10435-7.

## Introduction

Transposable elements (TEs), commonly referred to as jumping genes, hold great significance in eukaryotic genomes. Their widespread presence throughout the genome of parasitic worms is believed to significantly impact the structure and evolution of the host's genome [1]. TEs exhibit a diverse range of forms and boast a long evolutionary history [2].

TEs are DNA sequences that possess the ability to relocate within a genome. Based on their mode of movement within the genome, TEs can be broadly categorised into two main groups [3]. Class 1 elements, also known as Retrotransposons, have a unique mechanism of mobilization called "copy-and-paste." This involves an intermediate RNA that is reverse-transcribed into a cDNA copy, which is then integrated elsewhere in the genome [4]. Long terminal repeat (LTR) retrotransposons use an integrase to catalyze a cleavage and strand-transfer reaction for integration, similar to retroviruses [5]. Conversely, non-LTR retrotransposons, such as LINEs and SINEs, use a process called target-primed reverse transcription that links chromosomal integration to reverse transcription [6]. Class 2 elements, also known as DNA transposons, are mobilized via a DNA intermediate. This process can occur either through a 'cut-and-paste' mechanism or a 'peel-and-paste' replicative mechanism involving a circular DNA intermediate [7].

In most cases, the insertion of TEs does not provide any immediate advantages to their host organisms. Instead, these insertions tend to become established in a population due to genetic drift [8]. As time goes on, neutral point mutations can cause the degradation of these elements [2], rendering them unable to encode transposition enzymes or create new integration events [2].

With only a few exceptions, TEs can be found in the genomes of all eukaryotic organisms studied so far. The number of TEs in an organism's genome is closely linked to its size, and in some species, they can make up as much as 85% of the genome [8]. For example, the genome of the model organism *Caenorhabditis elegans* contains approximately 12% TEs [9].

Nematodes are incredibly versatile organisms that thrive in diverse environments. They can be found in soil, freshwater, seawater, hot springs, alpine regions, and even arctic areas, as well as in living and deceased organisms [10]. The Nematoda phylum is divided into five Clades, ranging from Clade I to Clade V, which are differentiated by their small subunit ribosomal RNA sequences [11].

Plant-parasitic nematodes are placed in Clades I, II, and IV. Observing the distribution of parasitic species within and between these Clades, it has been proposed that parasitism of plants occurred on three occasions [12]. Nematodes have variable genome sizes and protein-coding genes, ranging from 38–700 Mbp and 10,228–27,465 genes respectively [13]. The non-coding part of the genome includes regulatory regions, introns, and repetitive elements. Studies have shown that repetitive elements, such as TEs, play a crucial role in nematode genome evolution [6, 14–16].

To fully comprehend the evolutionary processes that contribute to nematode diversity and the selection of specific traits, it is imperative to have a profound understanding of their genome. Thanks to the advancements in DNA sequencing, researchers are now able to sequence numerous nematode genomes from various clades, allowing for a comprehensive understanding of their genome's composition, architecture, and evolutionary dynamics.

TEs can affect nematode genomes in diverse ways. These elements have been known to cause mutations and polymorphisms, alter the genome structure, introduce new genes, amplify DNA sequences, regulate genes, rearrange exons, and rewire regulatory networks [16, 17]. As a consequence, TEs can drive diversification, adaptation, and speciation [18, 19].

Plant-parasitic nematodes are a diverse group of parasites that have evolved to infect a wide range of plant hosts, causing significant economic losses in agriculture worldwide [20]. They represent a major threat to global food security, with an estimated annual economic impact of $80 billion worldwide [21]. These nematodes are known to exhibit remarkable host diversity, reflecting their genomic diversity, and have evolved unique mechanisms to interact with their hosts [22].

The genomic diversity of plant-parasitic nematodes is a key driver of their interactions with hosts [23]. This diversity allows for the evolution of new host-parasite associations and adaptation to changing environments. TEs are one class of genomic elements that contribute to this diversity [24].

The focus of the present study is to explore the genomes of twenty-six distinct plant-parasitic nematodes that belong to nine different genera in clade IV. The study aims to gain a better understanding of the composition, types, and distribution of TEs in the genomes of these nematodes, and to determine their frequency and distribution patterns. Furthermore, the study aims to gain insights into the evolutionary patterns and mechanisms that govern the movement and spread of TEs across the genomes of these nematodes.

This study's findings reveal significant variations in the TE composition and diversity, the TE age distribution even within a single genus. The research also uncovers that nematode species often undergo lineage-specific expansion and contraction of TEs, indicating a dynamic evolution of these elements.

The TE age analysis provides a fascinating insight, revealing that DNA transposons constitute the majority of ancient TE insertions, which could be attributed to their ability to move between locations within the genome. In contrast, the more recent TE insertions have an unexpected origin from the Gypsy and Pao superfamilies of LTR retrotransposons. These superfamilies are widespread in the plant kingdom and can code for various proteins, including proteases that play a crucial role in protein degradation [25].

## Materials and methods

### Genomic data sets and data preprocessing

To create TE libraries, twenty-six plant-parasitic nematode genomes from nine genera (*Bursaphelenchus*, *Ditylenchus*, *Globodera*, *Heterodera*, *Meloidogyne, Subanguina, Radopholus, Pratylenchus* and *Aphelenchoides*) and one free-living nematode, *Panagrellus redivivus*, were downloaded from NCBI (https://www.ncbi.nlm.nih.gov) (Table S1) and Wormbase (https://parasite.wormbase.org/index.html) [13] (Table S1).

The accession numbers of the genomes used in the present study are given in the Availability of Data and Materials Section and Table S1.

To ensure the accuracy and quality of the genomes used in the present study, I took several steps. First, I used a program called blastn [26] with a cutoff of 1e-20 against the nt v5 database [27] to scan the genomes of all species for potential contamination. This step is crucial in removing any contaminated sequences from the genomes, as these can skew the results and lead to inaccurate conclusions. Results were manually checked, and contaminated sequences were removed from the genomes as described [28].

Next, I used BUSCO v5.4.7 [29] to assess the genomes' contiguity and quality. This program compares the genomes to the Nematoda data set, Nematoda Odb10 [29], and identifies single-copy core genes. For the present study, I considered only those genomes containing more than 50% of these single-copy core genes, which indicates the genome's high quality and completeness.

### Phylogenetic analysis

BUSCO core genes were searched in genomes with default options to infer phylogenetic relationships among nematode species. Each species’ resulting single and multi-copy core genes were merged and used to identify ortholog genes using OrthoFinder v2.5.1 [30] with the -M msa option. I employed mafft [31] for alignment and fasttree [32] for tree generation, both through OrthoFinder v2.5.1 [30]. The resulting phylogenetic tree was based on 212 orthogroups, each with at least 51.9% of species containing single-copy genes. *Panagrellus redivivus* was used as the outgroup.

### Construction of species-specific repeat libraries and TE annotation in the genomes

I employed automated annotation methods to establish TE libraries tailored to specific species. This entailed utilizing the -LTRStruct option in RepeatModeler v2.0.5 [33] to identify TEs and generate consensus sequences. To classify the sequences, I applied a reference-based similarity search approach by merging Dfam v3.8 (November 2023) [34] and RepBase libraries (RepBase Update 20140131) [35]. Ultimately, I incorporated the resulting TE libraries, with the aid of RepeatMasker v4.1.5 (https://www.repeatmasker.org), to annotate TEs present in the genomes. To identify the TEs that were not classified in each species, a detailed process of clustering was performed. This process involved the use of OrthoFinder v.2.5.1 [36] with the -d flag, which allowed for input of DNA and enabled the clustering of unclassified TEs. This clustering process provided valuable information regarding the identification of shared and species-specific TEs in nematode genomes. By analyzing the unclassified fraction of TEs, it was possible to gain a better understanding of the diversity of TEs present in these genomes and to identify those that are unique to specific species. Overall, the process allowed for a more comprehensive analysis of the TEs in nematode genomes and deepened our understanding of their evolution.

As a part of the research, a screening was performed to identify peptidases that may be encoded by the Gypsy superfamily transposable elements. To accomplish this, TransposonPSI (https://transposonpsi.sourceforge.net), a tool designed for identifying transposable elements in genomic or protein sequences, was used to screen proteins in nematode species. This method enabled a detailed analysis of the Gypsy superfamily TEs, which allowed for identifying potential peptidases encoded within. To better comprehend the protease-encoding capacity of Gypsy superfamily transposable elements (TEs) in nematode species, hmmscan v3.4 [37] was used to screen protein-encoding Gypsy superfamily TE elements in plant-parasitic nematodes (12 species) with available proteome files derived from genome annotation. The screening method was applied against the PFAM database v36 [38], facilitating the systematic and accurate identification of proteases within the Gypsy superfamily TEs. The screening process aimed to identify and analyse the proteases encoded by these elements that could play a significant role in nematode parasitism.

### TE age distribution

To ascertain whether plant-parasitic nematodes have accumulated or transposed transposable elements (TEs) recently or in the distant past, I utilised the Kimura 2-parameter (K2Pm) to generate age distributions within each species' genome. This involved measuring TE nucleotide sequence divergence as intra-family Kimura distances *K-values* [39], considering the rates of both transitions and transversions. *K-values* were calculated for all TE copies of each element to estimate the “age” and transposition history of TEs. I used specialised scripts, buildSummary.pl and calcDivergenceFromAlign.pl, implemented in the RepeatMasker package v4.1.5 (https://www.repeatmasker.org) on alignment files post-genome masking. The results were visualised using createRepeatLandscape.pl script in the RepeatMasker v4.1.5 package (https://www.repeatmasker.org). The rates of transitions and transversions were calculated for the alignments and then converted into Kimura distances using the equation K = -1/2 ln(1-2p-q) – 1/4 ln(1-2q), where q represents the proportion of transversion sites, and p represents the proportion of transition sites.

### Statistical analyses

To investigate the relationship between genome size and each class of TEs, including DNA, LTR, LINE, and SINE, and to gain insights into the contribution of different TE classes to genome size in nematode species, a multiple linear regression analysis was performed, on log-transformed data. This statistical analysis was carried out using the lm() function in R v4.3.2 [40], which allowed for the examination of the predictive power of each TE class for the genome size.

## Results

### Phylogenetic relationships in the nematodes

**Fig. 1:**
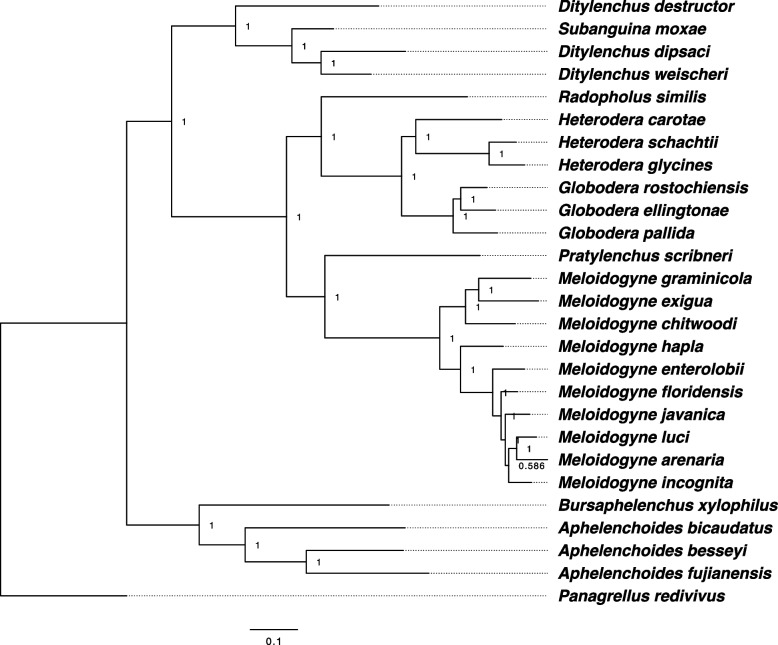
The phylogenetic relationships among nematode species. The phylogenetic tree was generated on 212 orthogroups, each with at least 51.9% of species containing BUSCO single and multi-copy core genes. *Panagrellus redivivus* was used as an outgroup species. Numbers on branches represent posterior probabilities

The phylogenetic analysis of nematodes has revealed interesting insights into the evolutionary relationships among different species (Fig. 1). Specifically, nematodes can be divided into two distinct clades based on their feeding behaviours. The phylogenetic tree is in line with the results that were previously published [41, 42]. The first clade includes the *Bursaphelenchus* and *Aphelenchoides* species, which are facultative migratory endoparasites (penetrate and feed within the host) and fungivores. Several *Aphelenchoides* species were reported to be ectoparasitic previously [43]. the *Bursaphelenchus* and *Aphelenchoides* species can use fungi as an alternative food source [44]. The second clade includes *Subanguina* and *Dityilenchus,* which are migratory endoparasites or fungivores [45], and placed in the basal and appear more distantly related to migratory and obligate endoparasitic nematodes (*Radopholus similis, Heterodera* spp., *Globodera* spp., *Pratylenchus* spp., and *Meloidogyne* spp.)*.* The findings suggest that the evolution of plant-parasitic nematodes is primarily influenced by their feeding behaviour. In other words, the way in which these nematodes feed may play a crucial role in their diversification.

### TE content diversity and contribution to nematode genome size

Plant-parasitic nematodes exhibit a wide range of TE content, which varies from 1.6% in *A. bicaudatus* to 55.7% in *D. dipsaci*. The TE content also differs between species in the same genus, as highlighted in Table 1.

**Table 1:** The genome size and percentage of TEs in the nematode genomes

Species	Genome Size (Mbp)	Genome Size (Mbp)^a^	Repeat %	DNA %	LTR %	LINE%	SINE %
*A.besseyi*	46.7	46.7	32.66	2.26	1.54	0.9	0.16
*A. bicaudatus*	46.4	45.6	1.64	0.04	0	0	0
*A.fujianensis*	143.8	114.9	38.67	3.03	4	0.51	0.37
*B. xylophilus*	78.2	78.2	27.88	6.34	1.22	0.08	0.43
*D. destructor*	110.8	109.7	19.26	2.89	0.68	0.29	0.02
*D. dipsaci*	227.2	222	55.7	10.32	2	0.55	0.05
*D.weischeri*	196.3	188.1	52.4	12.63	1.04	0.34	0.1
*G. ellingtonae*	105.9	104.3	28.05	3.87	1.48	0.21	0.18
*G. pallida*	122.9	118.8	20.16	2.48	0.84	0.09	0.08
*G. rostochiensis*	95.5	94.1	23.48	3.46	0.8	0.17	0.13
*H. carotae*	95.1	94.7	23.48	5.45	0.83	0.33	0.08
*H. glycines*	123.8	120.5	35.31	7.41	4.57	1.35	0.51
*H. schachtii*	179.2	172.4	35	5.26	4.63	0.79	0.15
***M. arenaria***	**283.8**	**283.2**	**52.4**	**6.6**	**5.97**	**0.82**	**0.06**
*M. chitwoodi*	47.4	47.4	18.57	3.94	1.73	0.04	0.01
***M. enterolobii***	**162.9**	**162.8**	**28.41**	**4.47**	**2.84**	**0.51**	**0**
*M. exigua*	42.1	42.1	18.92	4.15	0.42	0.09	0.02
*M. floridensis*	74.8	74.6	19.26	3.1	1.02	0.17	0.01
*M. graminicola*	41.5	41.5	11.26	0.94	0.35	0.02	0.27
*M. hapla*	52.6	52.6	13.74	1.49	1.52	0.34	0.08
***M. incognita***	**182.6**	**182.1**	**24.05**	**5.13**	**2.49**	**0.51**	**0.01**
***M. javanica***	**235.4**	**234.8**	**25.3**	**4.38**	**3.18**	**0.62**	**0.08**
***M. luci***	**209.1**	**209.1**	**30.85**	**6.73**	**4.88**	**0.47**	**0.23**
*P. scribneri*	227.2	226.4	29.03	4.58	3.97	0.53	0.09
*R. similis*	50.5	48.6	9.38	0.52	0.53	0.14	0.01
*S. moxae*	90.1	89.7	35.47	7.32	0.55	0.21	0.29

^a^After contamination was removed from genomes.

Bold names indicate the polyploid species.

DNA transposons dominated nematode genomes (Table 1). Of particular interest, the *D. weischeri* genome displayed a higher number of expanded DNA transposons when compared to other nematode species (Table 1). On the other hand, the *M. arenaria* genome showed expansion in LTR retrotransposons. Other retrotransposons, such as LINE and SINE, were more prevalent in *H. gylcines* (Table 1).

The statistical analysis (see the method) revealed that the DNA transposons significantly contribute to the overall genome size (p = 0.00455). On the other hand, the contribution of other TE classes, such as LTR, LINE, and SINE, was not significant, with p-values of 0.07744, 0.14651, and 0.88360, respectively.

### The TE diversity has evolved in different lineages

**Fig. 2:**
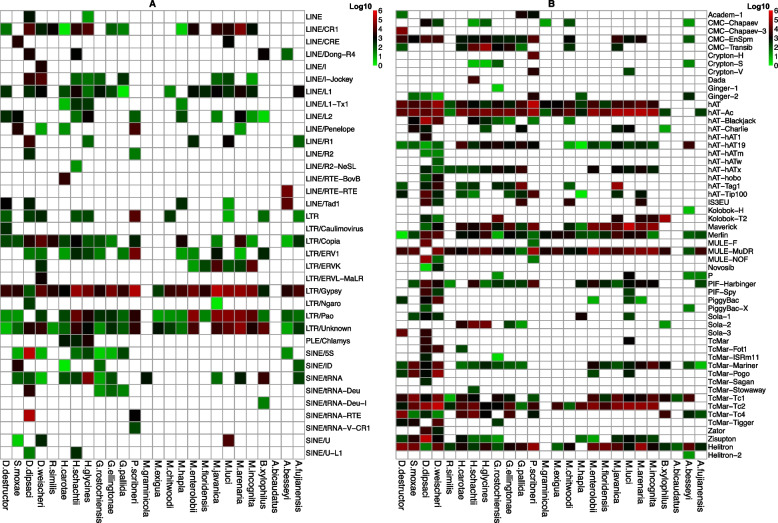
The diversity of TE superfamilies in the nematode species. **A** The Class I TEs and **B** Class II TEs (DNA transposons). The filled cells show the presence of TE superfamilies, and the colour gradient depicts the TE copy number. Conversely, empty cells indicate that the corresponding TE superfamilies are absent in the nematode species

To further analyse TE content and distribution in nematodes, levels of each TE superfamily were calculated. Although the distribution of TE superfamilies varied between nematodes, the study found that LTR retrotransposons, such as Gypsy and Pao (Fig. 2A) and DNA transposons, such as hAT and hAT-Ac, MULE-MuDR, Maverick, Merlin, and TcMar (Fig. 2B), were widespread and among the most predominant TE superfamilies in the nematodes.

The content and diversity of TEs varied significantly among different lineages, and even within the same lineage. For instance, in the *Meloidogyne* lineage, the percentage of TE content ranged from 11.26% to 52.4% (Table 1). Additionally, new TE families emerged in the inner clades of the phylogenetic tree (Fig. 1). TE families DNA/TcMar-Tc2 and DNA/HAT were absent in the *Bursaphelenchus* and *Aphelenchoides* lineage (Fig. 2B).

The results indicated that these lineages acquired new TE families during genome evolution. The distribution of TE families was specific to particular lineages. For example, DNA/Crypton-H was found only in *Pratylenchus* species (Fig. 2B). Another example of lineage-specific TE family acquisition is PLE/Chlamys, seen only in the *Heterodera* lineage.

Interestingly, the TE family PLE/Chlamys exhibits remarkable diversity even within the same genus. Moreover, the presence or absence of DNA/PIF-Harpinger varies among these species. Notably, *M. graminicola*, *M. exigua*, and *M. chitwoodi* lack this family, suggesting a possible loss during genome evolution.

The study found that the DNA/Ginger-1 subgroup was exclusively present in the *Globodera* lineage, and no other lineages showed its presence. Moreover, the DNA/Kolobok-T2 superfamily was specific to the *B. xylophilus* lineage and was not found in other lineages, such as the *Aphelenchoides* lineage in the same clade. The absence of the DNA/Sola-1 superfamily in *D. destructor*, a species belonging to the *Ditylenchus* and *Subanguina* clade, was noteworthy, as this family was present in other species within the same clade. It is worth mentioning that some *Meloidogyne* species showed the absence of LINE/L1 and LINE/L2, despite belonging to the same lineage. These observations indicated high TE diversity within the same clade and lineage (Figs. 1 and 2).

Certain TE superfamilies were exclusively identified in a specific nematode species. This observation was noted in distinct DNA transposons, Kolobok-H in *A. besseyi*, Dada in *H. schachtii*, (Fig. 2B) and specific LTR retrotransposons such as Caulimovirus in *D. destructor,* and ERVL-MaLR in *D. dipsac*i (Fig. 2A). Additionally, this was observed in non-LTR retrotransposons such as LINE/RTE-BovB in *H. carotae*, LINE/R2-NeSL in *H. schachtii* and SINE/tRNA-Deu-I in *B. xylophilus* (Fig. 2A). These superfamilies were absent in other nematodes (Fig. 2A).

#### Polyploid genomes typically contain a greater number of TEs compared to diploid genomes

Polyploidy, the condition of having multiple sets of chromosomes, is uncommon in animals compared to plants. However, a recent study was conducted to understand the genomic basis for the evolutionary success of three parasitic root-knot nematodes from the genus *Meloidogyne*, namely *M. incognita*, *M. javanica*, and *M. arenaria* [46]. The study found that these species turned out to be polyploids [46]. Changes in ploidy can partly explain the significant differences in genome size observed among species [47]. This variation is mainly caused by the presence of various types of TEs [48]. TEs can play crucial roles in different processes, such as diversifying subgenome-specific chromosome sets before hybridization, influencing genome restructuring during rediploidization, affecting subgenome-specific regulatory evolution, and even providing opportunities for domestication and gene amplification to improve functionality [49].

The present study highlighted clear differences of TE content in the diploid and polyploid species of the *Meloidogyne* genus. Specifically, the Class I and Class II TE superfamilies were expanded in the polyploid species. This expansion is a result of the multiplication of TE copies in the genome, which can cause structural variation and contribute to the evolution of these species.

Notably, the polyploid species of *Meloidogyne*, such as *M. arenaria*, *M. luci*, *M. enterolobii*, *M. incognita* and *M. javanica*, have a higher TE content compared to their diploid sister species (Table 1).

The analysis of *Meloidogyne* species has revealed exciting findings regarding different types of DNA transposons across the genus. While some species exhibit the presence of PIF-Harbinger in a distinct clade, suggesting that this sequence was acquired later in their evolutionary history, other *Meloidogyne* species do not contain this sequence at all (Fig. 2B).

Moreover, the study examined the TE superfamilies that expanded in the polyploid species' genomes. The results showed that various TE superfamilies contributed to the expansion of the polyploid species' genomes. For instance, the hATx DNA transposon family was present in the polyploid species of the *Meloidogyne* genus, including *M*. *incognita*, *M*. *arenaria*, *M. javanica*, *M. luci*, and *M. enterolobii*, indicating that these species acquired this family of DNA at some point in their evolutionary history. In contrast, diploid Meloidogyne species do not contain this family of DNA. Additionally, the study identified the DNA transposons hat-Ac, TcMar-Mariner, Tcmar-Tc1-4 (Fig. 2B) and LTR retrotransposon LINE/CRE, LINE/L1, and LTR/ERVK (Fig. 2A) superfamilies that were expanded in the polyploid species. These expansions can result in genome size variation and contribute to the species' genetic diversity.

#### Plant-parasitic nematodes have high species-specific TE content

To better understand the unclassified fraction of TEs in nematode species (as listed in Table S2), the sequences were further clustered. This process involved grouping similar sequences together, based on their nucleotide sequence, to identify patterns and similarities that could provide insights into their function. The analysis involved processing a total of 21,125 unclassified sequences to identify shared and species-specific unclassified TEs. Out of these, 55.6% (11,753) were successfully assigned to 3194 orthogroups, while 44.4% remained unassigned (Table S3). Interestingly, none of the unclassified sequences were common among all species, indicating significant species specificity.

The study also revealed that the average number of TEs per species in an orthogroup was less than one in 99.7% of the orthogroups (Table S4). Furthermore, the distribution of nematode species in an orthogroup was uneven, with the majority of orthogroups (1083) containing only one species (Table S5).

Furthermore, nematode species shared very few unclassified TEs, with the highest number of overlapped TEs being found between *D. dipsaci* and *D. weischeri* (Table S6). Specifically, 480 TEs including LTRs were shared between the two species (Table S6). The study also found that the unclassified fraction of TEs greatly varied among nematode species, indicating a high level of diversity in TE composition across different species.

The study found that LTR retrotransposons were frequently present in both assigned (Table S7) and unassigned categories (Table S8), indicating their significant contribution to nematode genome evolution. This information is crucial for comprehending the range and diversity of TEs across various species and could offer insights into their potential role in genetic variation and evolution.

#### Gypsy elements encode a higher number of peptidases in polyploid genomes

The Gypsy elements of species from various lineages presented in the phylogenetic tree (Fig. 1) were scanned to determine if they encode proteins. The study found that the number of proteins encoded by these elements varies between species, ranging from 13 in *M. enterolobii* to 1282 in *M. arenaria* (Table 2).

**Table 2:** The number of proteins and peptidase encoded by the gypsy type transposable elements in nematode genomes

**Nematode Species**	**Number of Proteins (n)**	**PFAM Domain**			
		**gag-asp_proteas**	**Asp_protease_2**	**Asp_protease**	**Total**
*A. besseyi*	15	0	0	0	0
*B. xylophilus*	51	18	16	6	40
*D. destructor*	17	1	1	1	3
*D. dipsaci*	52	3	3	2	8
*G. pallida*	237	79	74	22	175
*G. rostochiensis*	16	7	6	1	14
*H. glycines*	147	30	19	7	56
*M. arenaria*	1282	278	261	119	658
*M. enterolobii*	13	10	10	9	29
*M. hapla*	76	29	29	22	80
*M. incognita*	203	85	84	32	201
*M. javanica*	492	136	125	61	322

The process of screening proteins in nematodes revealed that Aspartic proteases are the most commonly encoded elements in these species (Table 2).

The number of hits for these proteases varied significantly among different nematode species. The highest number of Aspartic proteases was detected in *M. arenaria*, while the lowest number was found in *A. besseyi*. Interestingly, even within the same genus, there were variations in the number of proteases detected. For instance, the *G. pallida* genome contains 175 proteases, whereas the *G. rostochiensis* genome has only 14 proteases.

Additionally, different PFAM domains were observed to exhibit variations in the number of Aspartic proteases. According to Table 2, the gag-asp_proteas PFAM domain was found to be the most dominant Aspartic protease domain in nematodes.

The present study reveals that the number of peptidases encoded by the Gypsy-type TEs is also higher in polyploid species of the *Meloidogyne* genus, as shown in Table 2. The abundance of TEs in these genomes partly explains the larger number of peptidases identified in polyploid *Meloidogyne* species. These findings suggest that the presence of TEs in the genome of *Meloidogyne* species may be a contributing factor to their parasitic success, as they have the potential to increase the number of genes and peptidases, which could enhance their ability to infect and parasitize host plants.

Overall, the study's findings shed light on the genetic variations and evolutionary history of the *Meloidogyne* genus. The study's results suggest that TE superfamilies played a significant role in the species' evolution, contributing to the genetic diversity and structural variation of the species' genomes.

The *Meloidogyne* genus is known for its intriguing parasitic success due to the polyploid species' composite genomes rich in TEs [46]. These TEs result from allopolyploidization events and promote functional divergence and plasticity between gene copies [46]. These polyploid *Meloidogyne* species have higher numbers of genes in comparison to their diploid sister species, as shown in Table S1.

#### The age distribution among nematode lineages varies greatly

The Kimura distance, a method for estimating the genetic distance between two DNA sequences, was used to evaluate the sequence divergence within the species-specific TE content. The *K-values* obtained from this analysis provide valuable information on whether the transposition events occurred recently or in the distant past. Lower *K-values* suggest that the events were more recent, while higher *K-values* indicate that they occurred in the ancient past.

**Fig. 3:**
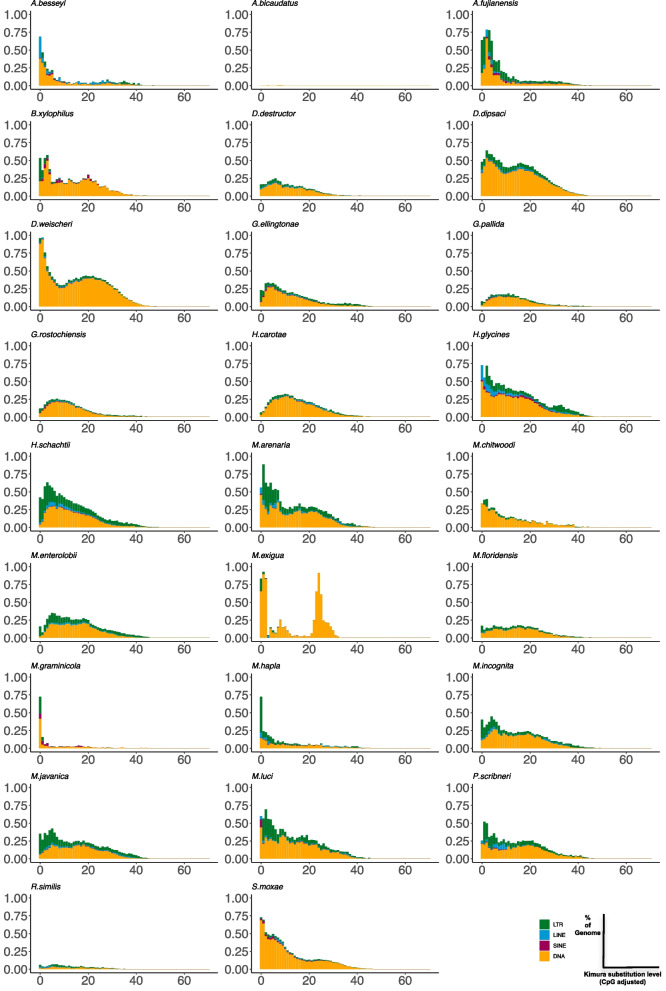
The Kimura distance-based copy divergence analysis of TE. The analysis of TE copy divergence using Kimura distance shows that clustering was conducted based on the distance. The distribution curve obtained from the Kimura distance-based analysis shows peaks on the right side, indicating younger TE fractions, while those on the left side represent ancient TE insertions. A small K-value suggests recent TE insertions, while a large K-value indicates ancient TE insertions

To further understand the TE age distribution within the genomes of different species, the Kimura distances based on their *K-values* were used to cluster the TE percentage in each species' genome. The clustering was depicted in Fig. 3, providing a visual representation of the TE content in each species' genome.

The analysis of TE copy divergence using Kimura distance shows that clustering was conducted based on the distance. Peaks on the right side of the distribution indicate younger TE fractions (small *K-values*), while those on the left side represent ancient TE insertions (large *K-values*). A small *K-value* suggests recent TE insertions, while a large *K-value* indicates ancient TE insertions (Fig. 3).

The significant variation in the age distribution of TEs among different clades and even in species of the same genus was detected (Fig. 3). It's worth noting that the LTR (Gypsy and Pao) and DNA transposon insertions have remained consistent during genome evolution in nematodes.

The age distribution of TEs exhibited significant differences among species within the same clade or lineage. In the *Bursaphelenchus* and *Aphelenchoides* clade *B. xylophilus*, it has been observed that the most recent TE activity in *B. xylophilus* is dominated by two types of LTR retrotransposons, namely Gypsy and Pao, as well as DNA transposons (CMC, Harbinger and hAT). These findings suggest that the TE activity in *B. xylophilus* has been primarily driven by these specific types of TEs in recent times.

*Aphelenchoides* species exhibit varying degrees of activity with regard to TEs (Fig. 3 and Tables S9-11). Specifically, in *A. besseyi*, young TEs were found, which included LTR (Gypsy and Pao), DNA transposons, LINE/L1, and RC/Helitron (Fig. 3 and Table S9). On the other hand, multiple RC/Helitron insertions were identified in *A*. *bicaudatus* (Fig. 3 and Table S9), whose TE content was the smallest among species and consisted of DNA transposons only (as shown in Table 1). It is important to note that TE activity in *Aphelenchoides* species is highly variable.

The analysis of the clade revealed that DNA transposons, specifically CMC and Harbinger, were relatively recent insertions. On the other hand, the result showed that the LTR retrotransposons, more specifically Gypsy and Pao, were also more recent insertions, but only in *Ditylenchus* species (Fig. 3, Tables S13-15) and not in *S. moxae* (Fig. 3, Table S34). These findings indicate a difference in the evolutionary history of transposons between the two nematode genera.

The TE age distribution in the *Radopholus* (Fig. 3 and Table S33), *Heterodera* (Fig. 3 and Tables S19-21) and *Globodera* lineage (Fig. 3 and Tables S16-18) had a uniform shape. The DNA transposons MCM and Harbinger were found in all three lineages. More recent TE insertions were observed on LTR retrotransposons (Gypsy and Pao) in the *Heterodera* lineage (Fig. 3 and Tables S19-21). Interestingly, recent Gypsy and Pao insertions were more frequently observed in *H. schachtii* and *H. glycines*, located in the inner clade and performed a sister clade, than in *H. carotae* (Fig. 3 and Tables S19-21).

The age distribution of TEs varies greatly between the *Pratylenchus* and *Meloidogyne* clades, as depicted in Fig. 3. In *Pratylenchus* species, multiple peaks were observed in the TE age distribution, indicating the presence of TEs with different ages (Fig. 3 and Table S32).

#### Transposition events tend to occur at an older age in polyploid genomes compared to diploid genomes

The variation in TE age distribution showed a clear pattern among nematode spcies (Figs. 3 and Tables S22-31).

The TE insertions were relatively older in the polyploid species compared to the diploid species. younger in the diploid species than, namely *M. graminicola*, *M. exigua*, and *M. chitwoodi* compared to The TE age dates back to in the polyploid *Meloidogyne* species (Fig. 3 and Tables S22-31). Those include *M*. *enterolobii*, *M. floridensis*, *M. javanica*, *M. luci*, *M. arenaria*, and *M. incognita*, belong to a sister clade to the diploid species and possess more ancient TE insertions (Fig. 3, Table S24, Table S26 and Tables S28-29).

Although LTR retrotransposons such as Gypsy and Pao, and DNA transposons, especially CMC, Harbinger, and hAT, are common TE insertions in these species, LTR transposons were found to be more frequently inserted TEs in the polyploid genomes (Fig. 3, Tables S22-31).

Polyploid species have been observed to contain a higher number of peptidases encoded by the Gypsy TEs than diploid species (Table 2). This correlation has been found to be due to the presence of intensive LTR retrotransposons, specifically Gpysy. These findings suggest that the origin of peptidases in polyploid species can be partly attributed to the presence of these retrotransposons.

Moreover, the patterns of TE insertion also differ between polyploid and diploid genomes. The TE insertion patterns in polyploid genomes have been observed to be more diverse than those in diploid genomes (Fig. 3, Tables S22-31). This observation suggests that polyploidization could result in the accumulation of more mutations and structural variations in the genome, leading to higher genetic diversity and adaptation potential in polyploid species.

## Discussion

### High TE diversity and species-specific TE content in nematode lineages

TEs are a crucial factor in shaping the genomes of different species. Previous studies have focused on newly sequenced species and only a few plant-parasitic nematodes [50–53], but with the help of Next Generation Sequencing technologies, more plant-parasitic species have been sequenced and annotated, leading to improved genome assemblies available at Wormbase [13]. As a result, a wealth of genome data on plant-parasitic nematodes has accumulated, providing new opportunities for genome-wide comparative studies. In this study, a great number of plant-parasitic nematode species from diverse taxonomic groups were investigated to identify and compare TEs between nematode species. I evaluated the results based on TE abundance, diversity, activity, and evolution in nematode species.

In this study, a noteworthy disparity in the quantity of TEs was discovered in nematodes. Moreover, this variation was observed even among species of the same genus. For instance, the *M. grammicola* genome constitutes only 11.26% of TEs, while the *M. arenaria* genome comprises 52.4% TEs. It is worth mentioning that the *M. grammicola* genome is the smallest in the *Meloidogyne* lineage. On the other hand, incomplete genomic coverage of TE hinders the accurate estimation of this variability. This problem is exacerbated when genomes are sequenced using short-read technology [54], as it is difficult to accurately reconstruct repeat regions that are longer than the insert size. This, in turn, leads to assembly errors and artefacts [55]. Therefore, it is important to exercise caution when interpreting genome size and TE content estimates and to consider the potential impact of incomplete genomic coverage on these estimates. The approach utilized for detecting and constructing TE libraries in plant-parasitic nematodes in this study was meticulously designed to minimize errors and generate dependable and resilient outcomes. This approach provides a solid foundation for inferring TE diversity and evolution in these nematodes with utmost confidence.

The study also found that DNA transposons are significant predictors of the genome size in the nematodes, indicating that DNA transposons are a crucial component of plant-parasitic nematode genomes. DNA transposons are the primary TEs in nematode genomes, which explains the diversity in TE content and variation in genome size. Additionally, the study revealed that DNA transposons in some species, particularly those with higher TE content, appeared to have recent transposition activity. The TE age and divergence analysis further supported this finding.

The study conducted on nematodes has revealed the prevalence of unclassified LTR retrotransposons in both the assigned and unassigned categories. This finding has significant implications for nematode evolution, as these genetic elements are known to play a vital role in the genomic plasticity of many organisms [24, 56, 57]. The presence of LTR retrotransposons in nematodes suggests that these genetic elements may contribute to the adaptability and diversity of nematode populations. These findings shed light on the molecular mechanisms underlying nematode evolution and open up new avenues for further research in this field.

Previous studies indicate that the proportion of TE content can differ within clades or lineages, even among species belonging to the same genus. This has been observed in various invertebrates, such as nematodes [41] and arthropods [58].

An exciting discovery was found regarding TEs in specific clades or lineages. Certain TEs were either present or absent, possibly due to their extinction or acquisition during the evolution of different nematode genera. The loss of TE superfamilies could occur in a few different ways, either from independent losses of multiple TEs belonging to the same superfamily or from a single loss of a TE superfamily if it was only a copy in the genome [58]. Additionally, specific TE superfamilies were only present in one species and absent in others within the same genus. This could suggest that these TE superfamilies were transferred from the host of the nematode species or through interactions with viruses or bacteria via infection or mutualism.

The occurrence and behaviour of TEs in the genomes of plant-parasitic nematodes vary, exhibiting active and inactive phases during their lifecycle. The age distribution results suggest that many of these nematodes experience more frequent insertions of LTR retrotransposons, particularly from the Gypsy and Pao superfamilies. The incorporation of new TEs may benefit these species and influence their genome. The newly acquired Gypsy elements could result from a horizontal transfer event or the evolution of a unique Gypsy lineage from an ancestral Gypsy element through genetic mutations. Gypsy retrotransposons are commonly present in plant genomes and encode several protein families, including proteases, integrase, reverse transcriptase, and ribonuclease [25]. Proteases, specifically, play a critical role in host-parasite relationships [59].

### The LTR retrotransposons are involved in plant-parasitism in the nematodes

Aspartic proteases are one of the protease families that show diverse functions in plant-parasitic nematodes. They are involved in several processes during the nematode's life cycle, including infection [60] and feeding [61].

The current study has demonstrated that the Gypsy TEs contain genes that encode Aspartic proteases. The recent insertion of these TEs in plant parasitic nematode genomes suggests that these TEs may be involved in parasitism during nematode-host interactions. The Aspartic proteases encoded by these TEs could play a crucial role in the nematode's ability to infect plants and evade the plant's defence system.

It is plausible that the recent acquisition of Gypsy retrotransposons is one of the major components of parasitism and is closely linked to adaptive evolution in plant-parasitic nematodes.

### The insertion of TEs is not evenly distributed throughout the genomes

The TEs have been studied extensively, and it was found that their age distribution varies significantly among different clades and even in species of the same genus. The activity of TEs is not uniform across different clades and species. LTR (Gypsy and Pao) and DNA transposon insertions have remained consistent and highly active and showed multiple insertions in nematode genomes during genome evolution. This suggests that LTR and DNA transposon insertions have significantly influenced nematode genome evolution.

The newly discovered TE insertions, originating from these superfamilies, could have an impact on the gene expression and/or function of the host organism, which could further influence its evolution. These findings provide insights into genome evolution and diversification mechanisms in plant-parasitic nematodes.

This study provides a deeper understanding of the genetic makeup and evolution of nematodes and highlights the importance of studying these elements for a comprehensive understanding of their biology.

The age distribution of individual TE superfamilies provides clear evidence of the lineage-specific burst-like TE proliferation mode observed in plant-parasitic nematodes.

Overall, the findings suggest that TE activity is a dynamic process that is not uniform across different clades and species. These findings have significant implications for our understanding of the evolution of plant-parasitic nematodes and parasitism.

### Supplementary Information

Supplementary Material 1.

Supplementary Material 2.

Supplementary Material 3.

Supplementary Material 4.

Supplementary Material 5.

Supplementary Material 6.

Supplementary Material 7.

Supplementary Material 8.

Supplementary Material 9.

## Data Availability

The genomes used in the current study are available at https://www.ncbi.nlm.nih.gov and https://parasite.wormbase.org/index.html. The accession numbers of genomes are *Aphelenchoides bicaudatus* (GCA_024699845.1), *Aphelenchoides besseyi* (GCA_024699855.1), *Aphelenchoides fujianensis* (GCA_024699865.1), *Bursaphelenchus xylophilus* (GCA_904067135.1), *Ditylenchus destructor* (GCA_001579705.1), *Ditylenchus dipsaci* (GCA_004194705.1), *Ditylenchus weischeri (*GCA_029231635.1), *Globodera ellingtonae* (GCA_001723225.1), *Globodera pallida* (GCA_000724045.1), *Globodera rostochiensis* (GCA_900079975.1), *Heterodera carotae* (GCA_024500135.1), *Heterodera glycines* (GCA_004148225.1), *Heterodera schachtii* (GCA_019095935.1), *Meloidogyne arenaria* (GCA_003693565.1), *Meloidogyne chitwoodi* (GCA_015183035.1), *Meloidogyne enterolobii* (GCA_003693675.1), *Meloidogyne exigua* (GCA_018905775.1), *Meloidogyne floridensis* (GCA_003693605.1), *Meloidogyne graminicola* (GCA_002778205.1), *Meloidogyne hapla* (GCA_000172435.1), *Meloidogyne javanica* (GCA_003693625.1), *Meloidogyne incognita* (GCA_900182535.1), *Meloidogyne luci* (GCA_902706615.1), *Pratylenchus scribneri* (GCA_030063045.1), *Radopholus similis* (GCA_013357305.1), *Subanguina moxae* (GCA_000981365.1). The datasets generated and/or analysed and codes used for analyses during the current study are available on GitHub https://github.com/mehmetdayi/TE_PPN.
