# Supplementary material for: Diversity and evolution of transposable elements in the plant-parasitic nematodes
Source: BMC Genomics. 2024 May 23;25:511. doi: 10.1186/s12864-024-10435-7 (PMC11118728; doi:10.1186/s12864-024-10435-7)
Supplement: Supplementary file 2 — Supplementary Material 2. [file 12864_2024_10435_MOESM2_ESM.docx]

| **Species** | **Number of Elements (n)** | **Length Occupied**  **(Mbp)** | **%**  **in the Genome** |
| --- | --- | --- | --- |
| *A.besseyi* | 4541 | 2,20 | 4,70 |
| *A. bicaudatus* | 3455 | 0,52 | 1,13 |
| *A.fujianensis* | 66466 | 33,35 | 29,01 |
| *B. xylophilus* | 67868 | 12,47 | 15,93 |
| *D. destructor* | 108039 | 15,02 | 13,68 |
| *D. dipsaci* | 569213 | 91,61 | 41,26 |
| *D.weischeri* | 497592 | 70,22 | 37,33 |
| *G. ellingtonae* | 147712 | 20,50 | 19,63 |
| *G. pallida* | 139731 | 16,78 | 14,12 |
| *G. rostochiensis* | 128297 | 15,47 | 16,43 |
| *H. carotae* | 107823 | 13,26 | 14,00 |
| *H. glycines* | 123352 | 21,42 | 17,77 |
| *H. schachtii* | 203569 | 35,38 | 20,52 |
| *M. arenaria* | 247340 | 43,09 | 15,21 |
| *M. chitwoodi* | 18731 | 3,39 | 7,15 |
| *M. enterolobii* | 187190 | 27,82 | 17,09 |
| *M. exigua* | 6640 | 2,37 | 5,64 |
| *M. floridensis* | 65095 | 8,45 | 11,31 |
| *M. graminicola* | 5322 | 1,10 | 2,65 |
| *M. hapla* | 19416 | 3,28 | 6,24 |
| *M. incognita* | 161423 | 23,20 | 12,73 |
| *M. javanica* | 240668 | 32,30 | 13,75 |
| *M. luci* | 166126 | 28,17 | 13,47 |
| *P. scribneri* | 155735 | 37,40 | 16,52 |
| *R. similis* | 28504 | 3,07 | 6,32 |
| *S. moxae* | 188918 | 23,76 | 26,46 |

**Table S2.** **The Distribution of Unclassified Fraction of Transposable Elements in the Plant-Parasitic Nematodes.**
